# Rice Husk Ash-Derived Silica Nanofluids: Synthesis and Stability Study

**DOI:** 10.1186/s11671-016-1726-9

**Published:** 2016-11-15

**Authors:** Zhiliang Zhang, Wenxiu He, Jianzhong Zheng, Guangquan Wang, Jianbing Ji

**Affiliations:** Zhejiang Provincal Key Laboratory of Biofuel, College of Chemical Engineering, Zhejiang University of Technology, Hangzhou, 310014 China

**Keywords:** Rice husk ash, Nanofluids, Silica nanoparticles, Stability

## Abstract

Nanofluids, colloidal suspensions consisting of base fluids and nanoparticles, are a new generation of engineering working fluids. Nanofluids have shown great potential in heat/mass transfer applications. However, their practical applications are limited by the high production cost and low stability. In this study, a low-cost agricultural waste, rice husk ash (RHA), was used as a silicon source to the synthesis of silica nanofluids. First, silica nanoparticles with an average size of 47 nm were synthesized. Next, by dispersing the silica nanoparticles in water with ultrasonic vibration, silica nanofluids were formed. The results indicated that the dispersibility and stability of nanofluids were highly dependent on sonication time and power, dispersant types and concentrations, as well as pH; an optimal experiment condition could result in the highest stability of silica nanofluid. After 7 days storage, the nanofluid showed no sedimentation, unchanged particle size, and zeta potential. The results of this study demonstrated that there is a great potential for the use of RHA as a low-cost renewable resource for the production of stable silica nanofluids.

**Graphical Abstract:**
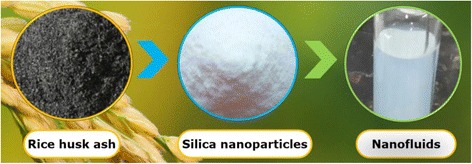
Rice husk ash was used as a low-cost renewable resource for production of silica nanofluids with high stability.

Rice husk ash was used as a low-cost renewable resource for production of silica nanofluids with high stability.

## Background

Nanofluids, a concept first proposed by Choi [1], are nanoscale colloidal suspensions containing nanoparticles. In the past decades, nanofluids have attracted more and more attention due to its extraordinary heat/mass transfer performance [2–5]. Nanofluids have demonstrated great potential applications in many fields such as automobiles [6], electronics cooling [7], industrial cooling [8], drug delivery [9], and CO_2_ absorption enhancement [10].

The application of nanofluids has significant prospects, but it still faces several challenges for future development [11]. Specifically, the low stability and high production cost of nanofluids are the major limiting factors [4, 11, 12]. Because of high surface energy, nanoparticles always have a tendency to coagulate automatically [13, 14]. Preventing the coagulation of nanoparticles is the primary issue for the application of nanofluids [15]. On the other hand, the currently available methods used for preparing nanofluids usually require expensive raw materials and sophisticated equipments, and thus leading to higher production cost [11, 12]. Low-cost production of stable nanofluids is one of the most promising directions for future research [11].

With the speedy development of green nanotechnology, there is a growing tendency to produce nanoparticles with renewable resources [16]. Rice husk, a low-cost agricultural residue, is abundantly available in rice-producing countries. Rice husk has a high calorific value (13–16 MJ/kg), and most of which is burned as fuel to generate energy, thus generating a significant volume of rice husk ash (RHA) [17]. If the RHA is improperly handled, it will become a tremendous waste and can potentially pollute the environment. Nowadays, there is an increasing demand for eco-friendly disposal and utilization of RHA. Many studies indicated that it was a promising low-cost candidate for preparation of silica nanoparticles [18–20]. Nano-sized silica can be used to form nanofluids with special interest because of its high specific surface area, excellent stability, high mechanical resistance, and possibility of reuse [21, 22]. However, to the best of our knowledge, most of the silica used for preparing nanofluids are commercially available nanoparticles. The commercially available silica nanoparticles are typically prepared by using a silica precursor as silicon source, such as silicon alkoxide (typically silicon tetraethoxysilane). However, the synthesis of silica precursors is usually energy intensive and associated with high cost, eco-hazardous, and unsustainability issues [18].

Generally, there are two primary methods for producing nanofluids [23]: (i) the one-step method, which represents the direct formation of nanoparticles inside the base fluids, and (ii) the two-step method, which means the preparation of nanoparticles and subsequent dispersion of nanoparticles in the base fluids. As compared to the single-step method, the two-step method is the most popular and economic process for the production of nanofluids [2], particularly, it is very suitable to prepare nanofluids containing oxide nanoparticles [14], such as SiO_2_, SnO_2_, CuO, and so on.

Kim et al. reported a sol–gel process for the synthesis of silica nanofluids [24]. TEOS (Tetra Ethyl Ortho Silicate) was used as precursor. The stability of silica nanofluids were determined by zeta potential analysis. However, the long-term stability information was not available. Fazeli et al. synthesized silica nanofluids with commercially available silica nanoparticles by the two-step method [25]. The nanofluids were stable for a period of 72 h without any visible settlements. Many other researchers also prepared silica nanofluids with commercial nanoparticles, but the stability results were not fully reported [26, 27].

**Fig. 1:**
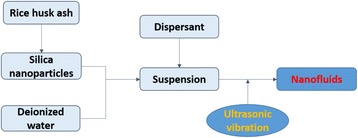
Flow diagram of the process for the preparation of silica nanofluids

In this work, RHA was employed as a low-cost silicon source for the synthesis of silica nanofluids by the two-step method. A flow diagram of the process was shown in Fig. 1. First, silica nanoparticles were synthesized using RHA as raw material. Then, silica nanofluids were formed by dispersing the silica nanoparticles in water with ultrasonic vibration. Silica nanoparticles were characterized by SEM, TEM, XRD, and FT-IR. The dispersibility and stability of silica nanofluids were also investigated in detail.

## Methods

### Reagents and Materials

Rice husk ash was from Ji’an of Jiangxi Province in China. Hydrochloric acid and anhydrous sodium carbonate were supplied by Sinopharm Chemical Reagent Co., Ltd (China). Sodium dodecyl benzene sulfonate (SDBS), sodium dodecyl sulfate (SDS), and polyethylene glycol (PEG-1000) were purchased from Aladdin in Shanghai (China). Sodium hexametaphosphate (SH) was obtained from Wenzhou Chemistry Material Factory (China). All chemicals were analytical grade.

### Preparation of Silica Nanoparticles

In a typical experiment, 100 g of RHA was added into 1000 ml of 1.0 M HCl solution and boiled for 2 h under stirring. The suspension was then filtered, and the solid residue was washed by distilled water to remove metallic ions. After that, the solid residue was dried in a ventilated oven at 120°C for 12 h.

After acid treatment, RHA (50 g) was put into a three-necked flask, and 250 ml of Na_2_CO_3_ solution (20 wt%) was added. The suspension was boiled with reflux condenser. After 4 h of vigorous stirring, the suspension was filtered and washed with 250 ml of hot water. Finally, the filtrate was transferred to another flask. CO_2_ was then introduced the filtrate. Stirring for 1 h, the resulting slurry (containing silica nanoparticles) was kept aging for 3 h and then filtered. The precipitate was washed by distilled water and was dried in a vacuum oven at 120°C for 24 h. The yield of silica was 71 ± 2%.

### Preparation of Silica Nanofluids

Silica nanofluids were prepared by the two-step method, and deionized water was used as base fluid. First, silica nanoparticles were synthesized using RHA as silicon source, as described above. Second, the silica nanoparticles were dispersed in water with the help of ultrasonic vibration, and thus forming silica nanofluids. Typically, 1 g of silica nanoparticles was dispersed in 100 g water by stirring. Next, 1 g of dispersant was dissolved in the suspension. Subsequently, the suspension was placed into an ultrasonic generator (JY92-IIN, Ningbo Scientz Biotechnology Co., Ltd, China) and sonicated for 2 h. The temperature was kept below 25°C.

### Characterization

The morphology of silica nanoparticles were observed by scanning electron microscopy (SEM, Hitachi S-4700) and transmission electron microscopy (TEM, Tecnai G2 F30). The column chart of the particle size distribution (PSD) was obtained using the Image-Pro 5.1 (Media Cybernetics, Inc.) software according to the SEM and TEM images. Zeta potential was measured using a dynamic light scattering instrument (Zetasizer-nano ZS90, Malvern). X-ray diffractometer (XRD) measurements were carried out using an X-ray diffractometer (X’Pert PRO, PANalytical). The results were recorded using diffraction from 15° to 60°, 2θ, at a scanning rate of 5°/min. Fourier transform infrared (FT-IR) spectra were recorded with a Nicolet model 6700 spectrometer (Nicolet Instrument Co., USA) in the range 400–4000 cm^−1^.

## Results and Discussion

### Preparation of Silica Nanofluids

**Fig. 2:**
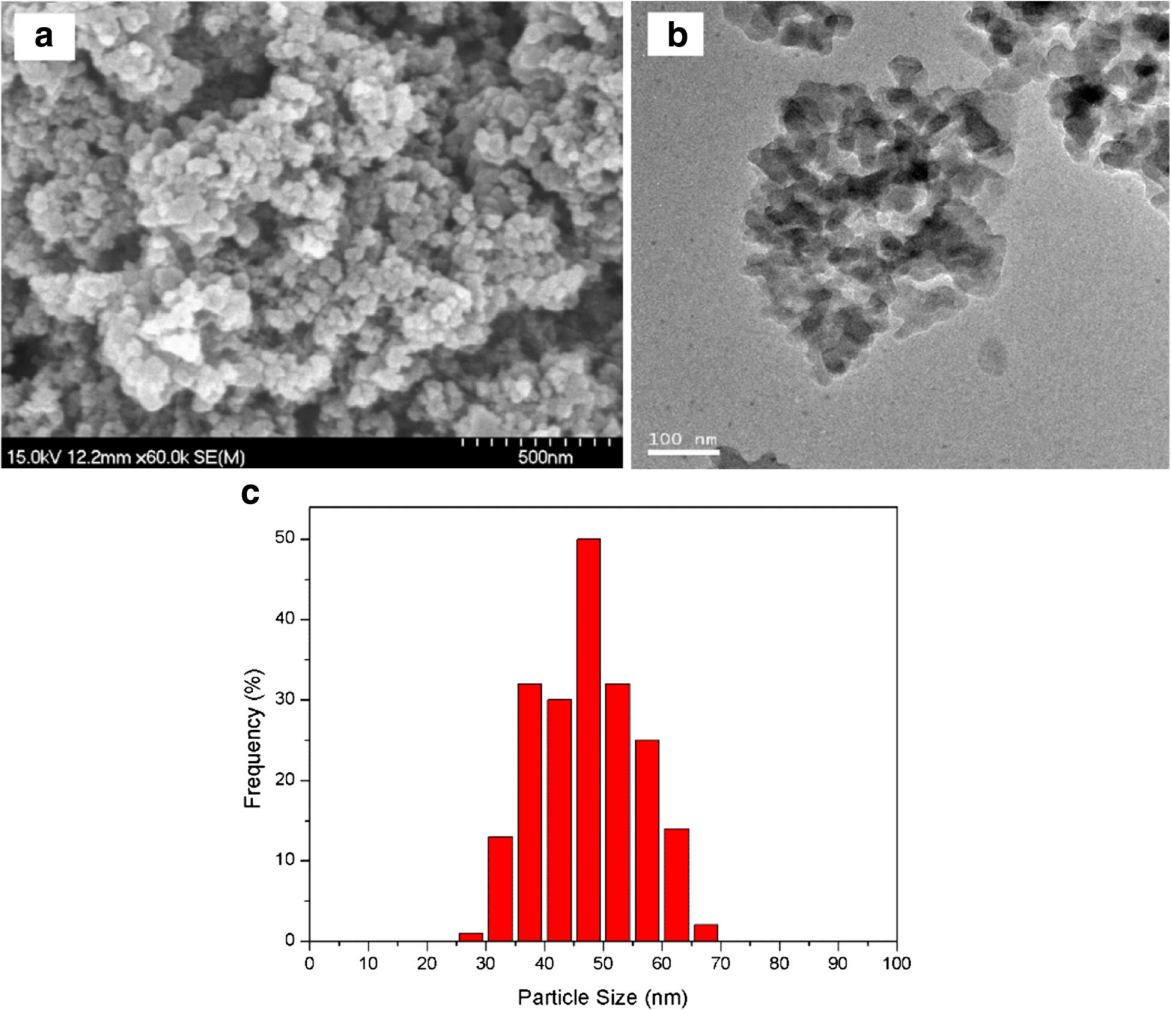
**a** SEM and **b** TEM images of silica nanoparticles prepared using RHA as a silicon source. **c** Particle size distribution of silica nanoparticles

**Fig. 3:**
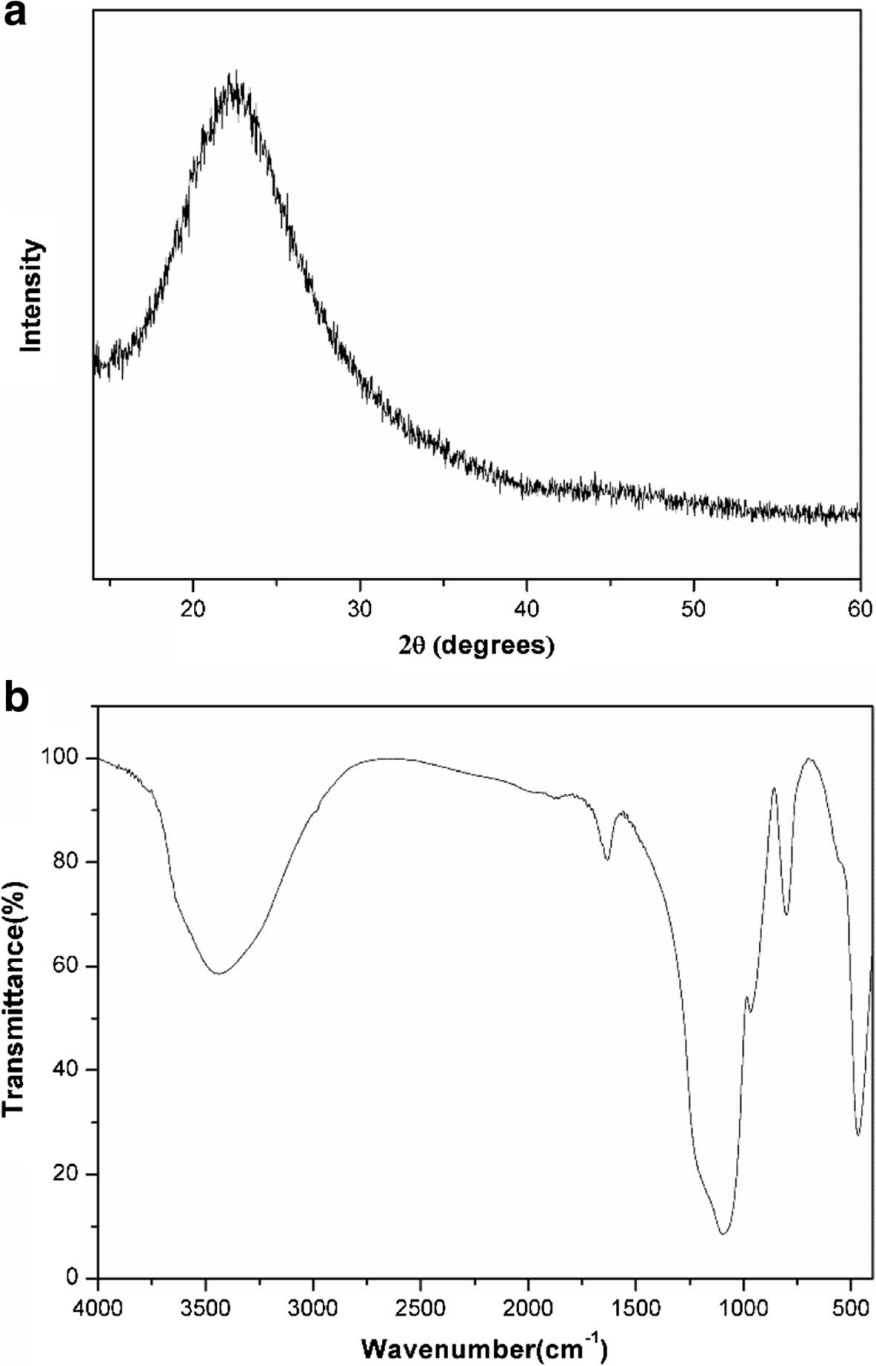
**a** XRD pattern and **b** FT-IR spectra of silica nanoparticles

In this study, the two-step method was applied to prepare silica nanofluids. First, silica nanoparticles were synthesized using RHA as a silicon source. Figure 2a shows the SEM image of the prepared silica nanoparticles. It could be seen that near-spherical silica nanoparticles with an average diameter about 50 nm were formed. The SEM result was further confirmed by TEM analysis (Fig. 2b). Figure 2c exhibits the particle size distribution of the silica nanoparticles. It is observed that the silica particles had relatively better dispersion. The average particle size was 47 nm. XRD pattern of the silica nanoparticles was shown in Fig. 3a, which shows a broad peak at 2*θ* angle of 22°, indicating the amorphous state of silica. FT-IR spectra of the silica nanoparticles were recorded by FT-IR spectrometer. As presented in Fig. 3b, the major chemical groups of silica are identified. The bands located at 467, 800, and 1095 cm^−1^ are ascribed to the consequence of stretching and bending vibrations of silica. The peaks at 3434 and 956 cm^−1^ are attributed to the Si–OH stretching vibration and bending vibration, respectively. The band at 1630 cm^−1^ belonged to H–O–H bending vibration of the adsorbed water. There were no other absorption peaks, which confirmed that the nanoparticles consisted of pure silica.

To form nanofluids, nanoparticles were usually dispersed in water with the help of intensive mechanical force agitation. Ultrasonication, a generally accepted mechanical technique, is widely used to improve dispersion behavior of nanofluids. Ultrasonication is very suitable for dispersing the highly entangled or aggregated nanoparticle samples [13, 28], especially nanofluids containing oxide nanoparticles [29].

**Fig. 4:**
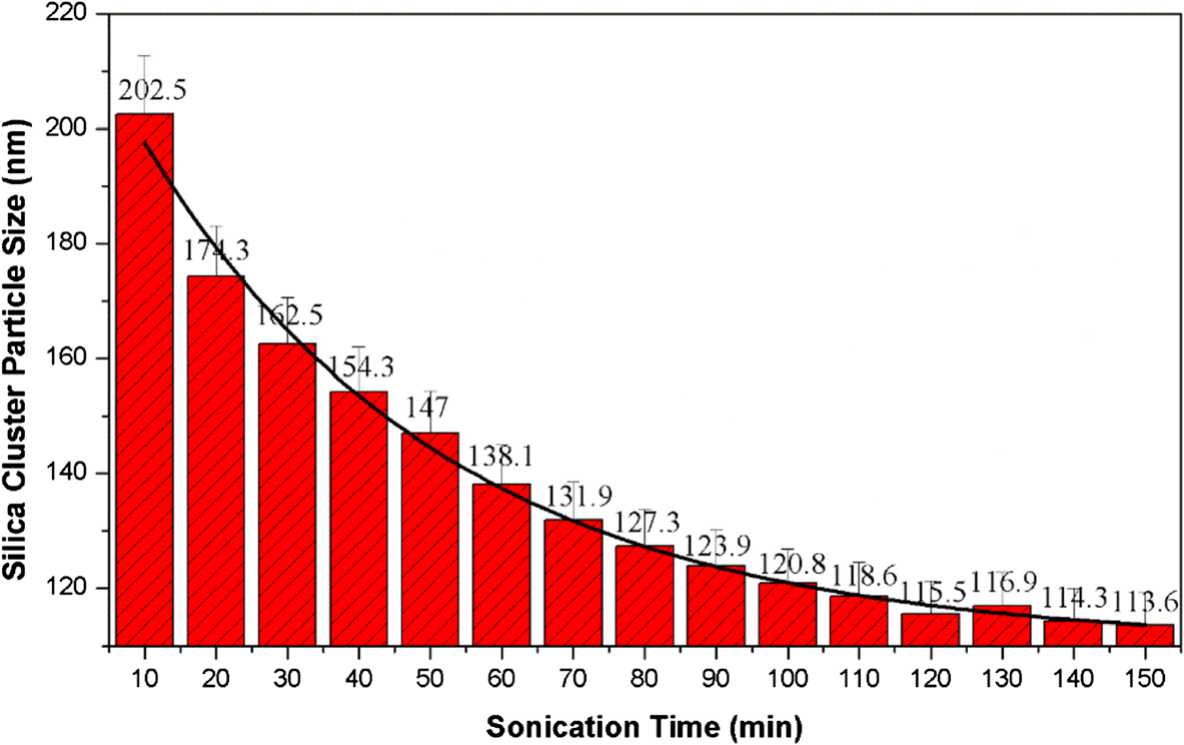
The silica cluster particle size as a function of sonication time (100 W, 20 Hz)

To investigate the effect of ultrasonic vibration on the dispersibility of silica nanofluids, 1 g of silica nanoparticles and 1 g of SDBS were added in 100 g of water and dispersed by an ultrasonic generator with an output power of 100 W (20 Hz). The relationship of the average silica cluster particle size and the sonication time was recorded using a dynamic light scattering instrument. As displayed in Fig. 4, the average cluster particle size was clearly decreased with the increasing of sonication time. This could be ascribed to the fact that silica clusters were gradually broken into small particles under intensive ultrasonic energy. After 120 min, however, the cluster particle size was 115 nm and basically unchanged, which indicated that the silica clusters cannot be effectively dispersed at the power of 100 W.

**Fig. 5:**
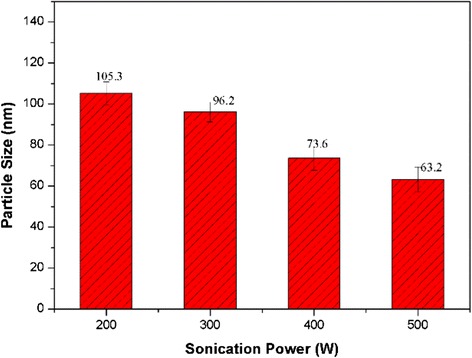
The average particle size as a function of sonication power (sonication time 120 min)

Enhancing the energy intensity of ultrasonic vibration is an effective way to improve the dispersibility of nanofluids. In order to obtain silica nanofluids with good dispersibility and stability, intensified ultrasonication power of 200, 300, 400, and 500 W (20 Hz) were further tested. The sonication time was set at 120 min for all cases. As exhibited in Fig. 5, with the enhancement of sonication power, the average cluster particle size was gradually decreased. At the sonication power of 500 W, the average particle size of the silica nanofluid was about 63 nm, which demonstrated that the silica nanoparticles had been completely dispersed in water, forming well-dispersed nanofluid.

### Influence of Dispersants on the Stability of Silica Nanofluids

The stability of nanofluids is very important for their practical applications, and it is closely related to nanofluids’ electrokinetic properties [13]. High-stable nanofluids can be formed with high surface charge density to provide sufficiently repulsive forces. The investigation of the electrophoretic behavior through measurement of the zeta potential is important for understanding the dispersion behavior of nanofluids [13, 28, 30]. Generally, nanofluids with high absolute zeta potential are electrically stabilized and have good stability, whereas those with low absolute zeta potentials are easy to coagulate or flocculate. It is widely accepted that nanofluids with the absolute zeta potential above 30 mV are physically stable.

**Fig. 6:**
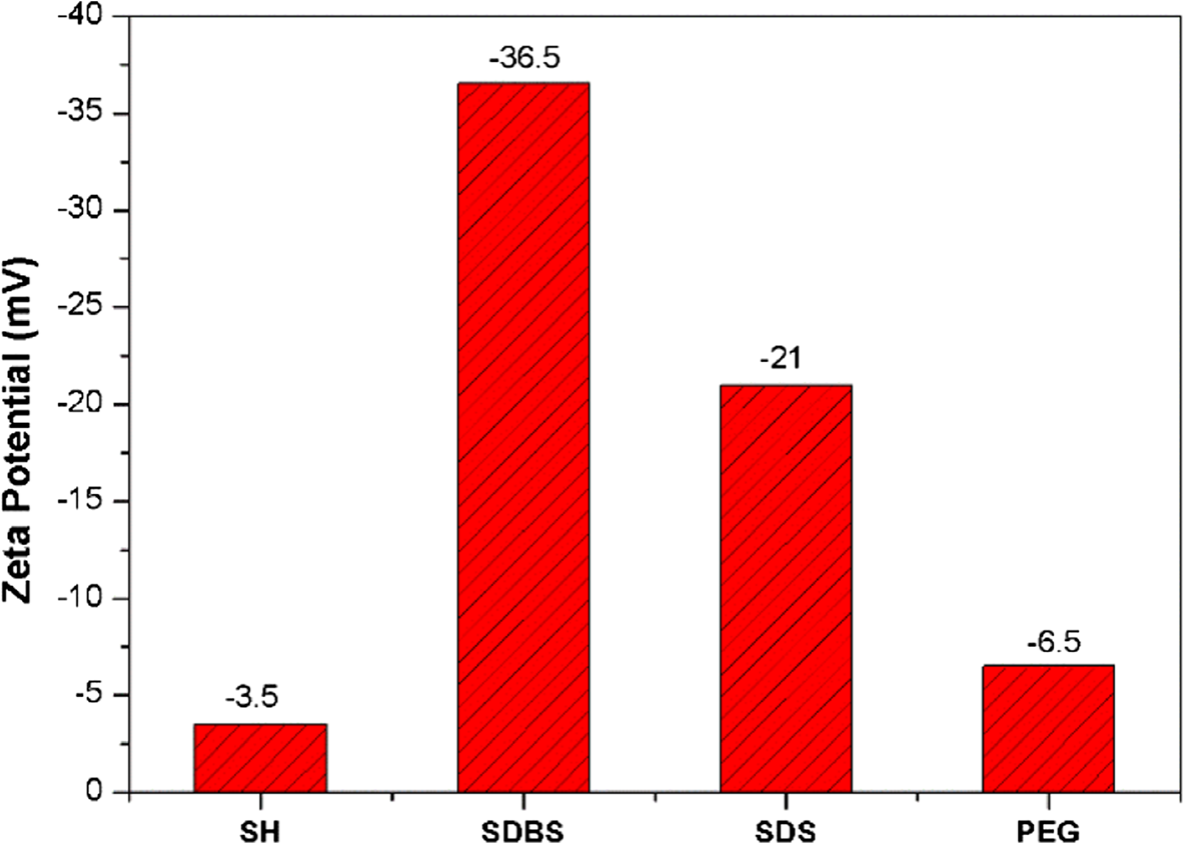
Influence of dispersants on zeta potential of silica nanofluids

Adding dispersants is an easy and economic way to improve the stability of nanofluids. Dispersants can adsorb on particle surface, and thus notably changing the electrokinetic characteristics of nanoparticles. In this study, four surfactants including sodium dodecyl benzene sulfonate (SDBS), sodium dodecyl sulfate (SDS), sodium hexametaphosphate (SH), and polyethylene glycol (PEG-1000) were tested as dispersants for stabilizing silica nanofluids. The concentration of the dispersants was set at 1.0 wt%. The solid concentration and pH of silica nanofluids were 0.5 wt% and 7.0, respectively. As shown in Fig. 6, the silica nanofluids were negatively charged with the tested dispersants. For SDBS, zeta potential was −36.5 mV, which demonstrates a stable system. While for the other three dispersants, the absolute zeta potential was smaller than 30 mV, stabilization fail. The plausible stabilization mechanism of SDBS is as follows: SDBS can partially ionize in water and thus generate negatively charged dodecylbenzene sulfonate. The anionic species could be adsorbed on the surface of silica nanoparticles, which leads to an electrostatic stabilization effect [13].

### Influence of SDBS Concentration on the Stability of Silica Nanofluids

**Fig. 7:**
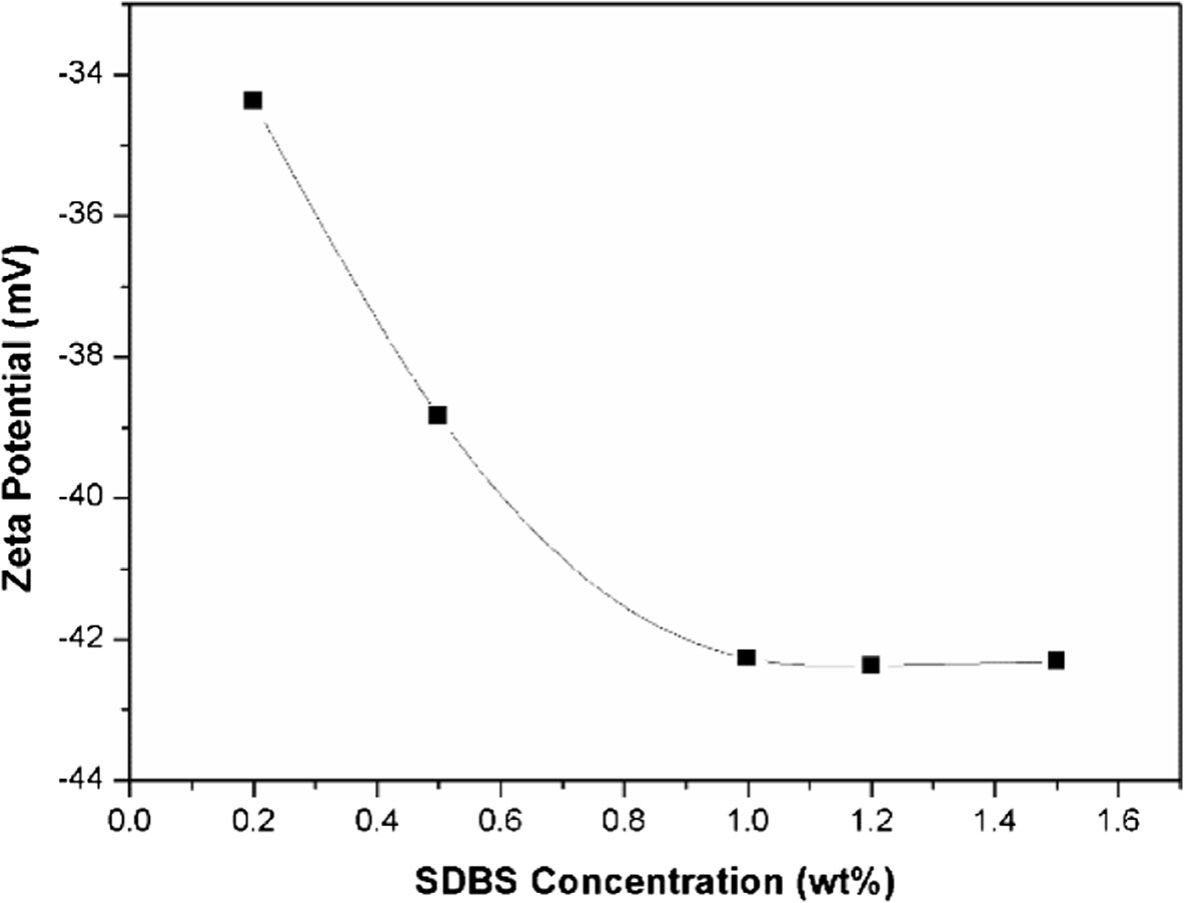
Influence of SDBS concentration on zeta potential of silica nanofluids

The influence of SDBS concentration on the stability of silica nanofluids are shown in Fig. 7. It can be seen that as the SDBS concentration increases from 0.2 to 1.0 wt%, the absolute zeta potential increases to a maximal value of 42.3 mV. However, further increasing SDBS concentration to 1.2 or 1.5 wt%, the absolute zeta potential was not increased. This may be attributed to the reasons that with the increase of SDBS, the anion groups pushed into the adsorbed layer causes the thickness of electrical double layer increases, thereby increasing the electrostatic repulsion among the particles [13]. When SDBS concentration reached to 1.0 wt%, an equilibrium state is reached between the repulsive force and van der Waals attractive force.

### Influence of pH on the Stability of Silica Nanofluids

**Fig. 8:**
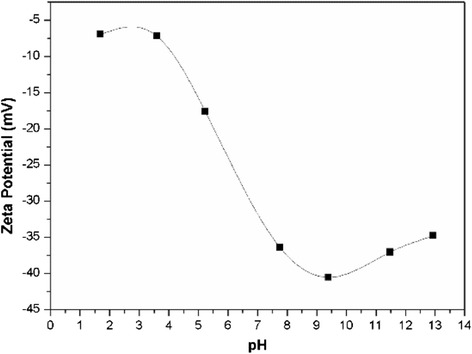
Influence of pH on zeta potential of silica nanofluids

Figure 8 shows the change of zeta potential as a function of pH. When the pH was below 3.5, the absolute zeta potential value was at the minimum. Due to the repulsive force is not sufficient to overcome the van der Waals attraction force, the dispersion stability is poor [31, 32]. As the pH is increasing, the absolute zeta potential value increases, the repulsive force becomes sufficient to prevent attraction and collision among particles caused by Brownian motion. At pH ≈ 9.5, the zeta potential value reached the maximum level of −40.5 mV, which demonstrated that the repulsive force is the strongest, and the dispersion stability of nanofluid is the best. Further increase of the pH to 11.5 and 13.0, however, the absolute zeta potential value decreased to 37.1 and 34.8, respectively. This fact could be explained by the following reasons: as the pH increases, the concentration of the pH adjustment reagent (i.e., NaOH) increases, which makes the compression of electrical double layer, thus resulting in a lower absolute zeta potential value [13].

### Long-term Stability of Silica Nanofluid

**Fig. 9:**
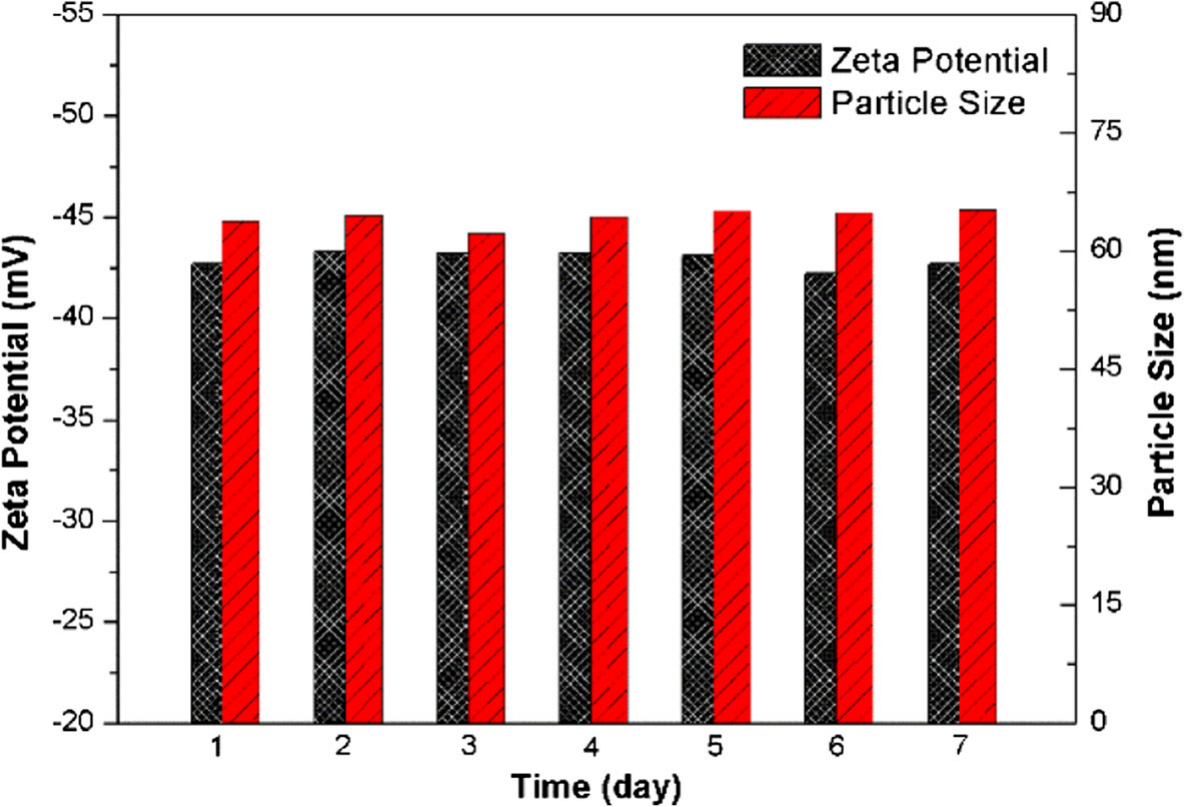
Zeta potential and particle size of silica nanofluid with storage time

Long-term stability is a crucial characteristic of nanofluids. The particle size analysis, zeta potential measurement, and visual observation were used for analyzing the nanofluid’s stability. Silica nanofluid (pH = 9.5, 1 wt% silica nanoparticles, 1 wt% SDBS) was prepared by ultrasonic vibration (sonication power 500 W, sonication time 2 h). As shown in Fig. 9, the particle size and zeta potential were basically unchanged during 7 days storage at room temperature. Moreover, no sedimentation was observed, which indicates a good stability of the silica nanofluid.

## Conclusions

Rice husk ash-derived stable silica nanofluids were prepared by the two-step method. Ultrasonic vibration was employed to disperse silica nanoparticles in water. Well-dispersed silica nanofluids could be obtained with the sonication power of 500 W. SDBS was a suitable dispersant for silica nanofluids. With 1.0 wt% SDBS, the absolute zeta potential could reach to a maximal value of 42.3 mV. The stability of silica nanofluids was also highly dependent on pH. At a pH of 9.5, the stability of nanofluid is the best. Long-term stability study indicated that nanofluid prepared at the optimal experiment conditions showed unchanged particle size and zeta potential during 7 days storage. Therefore, RHA is a promising low-cost renewable resource for the preparation of stable silica nanofluids.
